# Training the Next Generation: Developing Health Education Skills in Undergraduate Public Health Students at a Historically Black College and University

**DOI:** 10.3389/fpubh.2017.00274

**Published:** 2017-10-20

**Authors:** Krista Mincey, Tyra Gross

**Affiliations:** ^1^Department of Public Health Sciences, Xavier University of Louisiana, New Orleans, LA, United States

**Keywords:** skill development, public health, pedagogy, undergraduate, HBCU

## Abstract

With the looming workforce crisis, undergraduate public health students could be an important link in filling this demand. As public health continues to face challenges in the future, it is important that the future workforce is not only diverse but also trained in a manner that exposes them to real-world experiences that give them an opportunity to apply coursework to solve problems. This article outlines how a health program planning course was taught at a Historically Black College and University using assignments that promote active learning. Students were assessed on their ability to plan and implement a health activity based on a developed metric. Student and instructor reflections were collected from final assessments of the health programs by both groups. All elements of the course are discussed from course design, structure, assignments, and outcomes along with student and instructor reflections and lessons learned. Results suggest that including assignments focused on active learning are beneficial to helping students learn course material. As public health continues to change, more work needs to focus on teaching pedagogies that better prepare students to address future public health issues.

## Introduction

With the looming public health workforce crisis and efforts to increase diversity within public health, many schools and programs of public health are working to ensure that students are adequately prepared to meet current and future public health needs. Two of the responses that the Association of Schools of Public Health reported as ways to address the public health workforce crisis were (1) to increase public health educational capacity by providing certificate programs and increasing undergraduate education and (2) to increase the diversity of the public health workforce (1). Minority serving institutions, Historically Black College and Universities (HBCUs), or Hispanic-Serving Institutions are two avenues to achieve both of these endeavors toward addressing the public health workforce crisis. For the purposes of this paper, the focus will be on HBCUs.

The mission of all HBCUs is to educate African Americans. While the demographics of HBCUs might be changing as a reflection of the increasing diversity of the United States, the mission is still the same. Because of this mission, HBCUs are an underused resource to not only increase the public health workforce but to also increase diversity within the public health workforce (2). Currently, there are 138 accredited schools or programs of public health in the United States (3). Among this number, only seven programs are at HBCUs and none include a bachelor’s degree in public health (3).

The importance of HBCUs in increasing a diverse workforce is seen in their ability to graduate a large number of African Americans with science and engineering degrees (4). While HBCUs provide an environment that is beneficial to the success of many of their students, many times they lack the financial resources needed to provide students with experiences and other elements that may be beneficial to their success (4). Because HBCUs have continued to provide successful academic environments for their students despite a lack of resources, perspectives on teaching pedagogy at HBCUs are warranted.

As public health seeks to improve health equity by reducing health disparities, it is important to have a diverse public health workforce. Providing undergraduate public health degrees at HBCUs is one way to achieve this. Additionally, having undergraduates who are well-trained may be a way to address the looming public health workforce crisis (5). As the Council on Education for Public Health (3) continues to accredit more standalone baccalaureate programs of public health, perspectives on teaching pedagogy and student outcomes in baccalaureate programs is needed. Thus, the purpose of this article is to provide insight and thoughts on teaching work skills to undergraduate students in a health program planning course at a southern HBCU through the use of active learning. We believe this teaching of this course is innovative because there are few examples of how to prepare undergraduate public health students for the workforce; thus, our course example gives insight into the type of activities that might allow students to be more active learners who engage with and retain the information presented to them.

## Pedagogical Framework

Active learning is a teaching method focused on student engagement through the use of different classroom activities that require students to be engaged, to work with their peers, to apply current knowledge while gaining new knowledge to solve a problem (6, 7). Results on the effectiveness of this teaching technique in introductory and higher level courses is mixed (7, 8). However, active learning has been cited as a way for educators to teach medical students about the social determinants of health (9). Even with mixed findings, active learning has found promise in public health coursework with authors suggesting that courses using teaching methods such as active learning and focused on competency development are essential to public health core curriculum (6).

## Course Design

### Learning Objectives and Competencies

This course had seven learning objectives and two competencies. The competencies for this course come from the ten overall competencies for the degree program that were developed by the program’s founder. The learning objectives were also developed by the program’s founder. The competencies and objectives are listed below. Next to each objective, in parenthesis, you will see which assignment(s) addresses the objective.

#### Learning Objectives

1. Identify rationales for health promotion program planning to address primary, secondary, and tertiary health (group health program project).
2. Describe the application of behavioral theories in developing, implementing, and evaluating health promotion programs (group health program project).
3. Discuss the importance of health promotion planning in addressing health disparities (group health program project).
4. Utilize principles of health promotion planning to identify the needs of program participants (group health program project, health education presentation).
5. Identify ways to communicate health information to target populations (group health program project, health education presentation, skill demonstration presentation).
6. Identify ways to evaluate health promotion programs (group health program project).
7. Identify health promotion challenges and opportunities (group health program project).

#### Competencies

1. Apply theories, intervention frameworks, and or models to address public health concerns at the individual, group, and population levels.
2. Identify and apply key principles in program evaluation and applied public health research to assess the effectiveness of public health interventions.

### Course Description

Health Promotion Program Planning & Evaluation is a 2,000-level required course for undergraduate public health majors and minors. This course is taught as a traditional 50 min, 16-week semester course that is taught 3 days a week. This article discusses the teaching of the course during the fall 2016 academic term. For this course, students are required to work in groups to develop and implement a health program. In past sections, students have developed activities for their fellow college students around topics such as substance abuse and healthy eating. They have also worked with a community organization’s after-school program geared toward reducing obesity in elementary and high school children by developing activities around physical activity and preparing healthy meals.

### Course Structure

This course is setup so that students develop their health program over a period of weeks through different course assignments where they receive feedback from the instructors (Table 1). Because assignments start early in the course, students select their groups the first or second week of class. To assist students with understanding how to develop their health program, the instructors have the students work as a class on activities related to the material that is being covered. For example, when going over how to write goals and objectives, the students review examples, determine if the examples are right or wrong based on what they learned, and then develop goals and objectives as a class for a “fictitious” health program topic such as teen pregnancy. Engaging in this activity allows the students to see what they need to do to develop goals and objectives and how the goal and objectives for their health program need to be worded. Additionally, class time is given for groups to work on assignments and to ask the instructors for guidance on elements they might be confused about.

**Table 1:** Course structure.

Week	Topic	Assignments
1	Health promotion programs and health disparities	
	Working with communities	
2	Health theory	Article review
	Needs assessment	
3	Developing goals and objectives	
4	Program planning	Needs assessment due
5	Evaluation	
6	Implementation	Goals/objectives due
		Quiz
7	Health education presentations	Evaluation due
8	Health education presentations	Program plan due
9	Advocacy/marketing	Article review
10	Budgeting	Press kit due
11	Skill demonstration	Personal budget due
12	Skill demonstration	
13	Skill demonstration	
	Implement program	
14	Group presentations	Group paper due
15	Group presentations	
16	Group presentations	

For this course, the desire is always to have students work with a community group to develop health activities so that they are better able to engage the course material. Due to student schedules, this is sometimes not feasible if community groups need events to take place in the evenings or the weekend. Because of this issue and the large size of the class, it was decided that the class would plan a health program for their peers on campus. To help them focus their general topic, each group was assigned a health topic based on specific areas covered in the American College Health Association National College Health Assessment questionnaire. Two groups were assigned the topic of poverty, two groups were assigned mental health, and one group each was assigned alcohol, tobacco use, violence, obesity with a focus on nutrition, and obesity with a focus on physical activity.

## Course Assignments

To engage the students in the course with the material, activities and assignments were developed that would have students engaged in active learning. With most of the activities, students had to work in groups to apply the knowledge they learned in class and the textbook. Additionally, students had to draw on their own knowledge of being a college student to develop health activities for this target population that they believed would be effective. Below you will find descriptions of each assignment.

### Needs Assessment

So that students have a foundational basis for their health program, each group conducts a needs assessment for their target population around the given topic. Due to time constraints of the course, students use statistical reports from secondary data as the basis for their needs assessment. Groups provide a general overview of their target population’s health and then provide information on their specific topic. For example, they provide a general overview of college student health and then provide information on college students and physical activity.

### Goals/Objectives

After the needs assessment is completed, groups then develop a goal and SMART objective for their program based on the information in their needs assessment. Groups can have more than one objective, but they are only developing an activity around one objective. In addition to developing a goal and objective for their program, they must also provide justification for their goal and objective. For example, if their needs assessment talks about obesity and physical activity issues with college students, then their goal and objective should be focused around these issues.

### Program Plan

Once the goal and objective are complete, groups use this goal and objective to develop the health program that they will implement that will help them reach their objective. Each group provides details on how they will implement this program by giving full descriptions of the activity, which group member will do what, what day(s) the activity will be held, location, and time. This assignment usually takes the most time so more in class time is devoted to giving groups time to discuss their plans while getting feedback from the instructors before turning the assignment in.

### Press Kit

So that students gain some experience with how to market a program, each group develops a small press kit for their health program. Each press kit consists of a written 20 s public service announcement, a brochure that can be given out at their event, and two social media blurbs (Facebook, Instagram, Twitter) that can be sent out to advertise for their event. The public service announcement is placed on letterhead that includes each group’s logo. Additionally, the logo is used in the brochure and in the social media blurbs. For the brochure, groups have to make sure it’s at the appropriate reading level, and that they use pictures to help relay some of their points, and that the brochure is clearly printed in color and black and white. This assignment has evolved throughout the course. When the course was first taught, this assignment was not required. After realizing the importance of social media, the first author decided to add this to the course so students understand how to utilize social media for health purposes. Additionally, this assignment gives students an opportunity to understand social marketing on a basic level since the current public health curriculum doesn’t have a social marketing course.

### Evaluation Questions/Methods

While groups are not required to evaluate their health program, each group had to write up what questions they would ask to find out if their program was successful and what methods they would use to answer these questions. Groups had to make sure their evaluation questions lined up with their goal and objective and connected to the program that they implemented. Additionally, groups had to provide information on what type of data they would collect and why and how they would collect this data. Even though data were not required for this assignment, most groups provided statistics for their health program during their final presentation to show if their objective was reached.

### Health Education Presentation

To help students gain confidence in speaking in front of a group and to prepare them for implementing their health program, all students give a health presentation for the class. So that they are prepared for any situation that may occur when presenting in communities, students are not allowed to use technology (e.g., PowerPoint) for their presentation. They are graded on their engagement of the audience, their ability to relay their message clearly, and their ability to keep their eyes on their audience. Most students lack confidence in speaking in front of crowds; therefore, it is important to give them opportunities that allow them to be in real-life scenarios that may occur such as a technology failure while giving a presentation to a community or a professional audience.

### Skill Demonstration Presentation

Because educating a population on any health topic may involve developing skills in the population, students give a skill demonstration presentation to the class. Students can pick any skill they want, but they have to show the class how to do it and allow time for the some in the class or the whole class to demonstrate what they learned. Students are graded on engaging the audience, providing clear instructions, and allowing the audience to demonstrate the skill they gave instructions for. While students can use technology for this presentation, students didn’t use technology for this presentation. Examples of skill demonstrations have included how to tie a necktie, at home manicures, and American Sign Language.

### Metrics

For each assignment, students were given a set of guidelines to follow and were graded on how well their group followed the guidelines and provided the information needed. For the final presentation of the development and planning of their health program, groups were graded using a rubric developed by the first author. Groups were graded on their eye contact, ability to speak clearly, and have every group member participate in the presentation, organization of the presentation, and coverage of all topics (target population demographics; background on health issue; title, goal, objective, activity; program implementation, evaluation questions, and results, and how activity could have been improved).

The individual assignments of the health education and skill demonstration presentations were also graded using a rubric developed by the first author. For both presentations, students were graded on their eye contact, ability to speak clearly, organization, coverage of health topic, or how well they demonstrated the skill.

## Outcomes/Assessment

At the end of the course, each group does a presentation on their health activity. They provide background on their health topic and why the area (i.e., obesity) should be addressed, goal and objectives, health program, implementation, results, and final thoughts. With this presentation, both authors graded the students using the rubric mentioned in the metrics section. Additionally, each group member receives a grade from the other members of the group based on their input throughout the project, their attendance at meetings, and the completion of the work they were assigned. Examples of some health activities that groups developed during this course are below.

### Drinking Awareness Biz (D.A.B)

With this activity, group members did a short presentation on binge drinking facts to approximately 30 freshman men and women at the university through the housing department’s mentor program for freshmen. In addition to providing facts on binge drinking, they also provided students with ways to avoid drinking and binge drinking.

### Fight the Bite

For this activity, the group had a table in the student center for 2 days where students came and played games testing their nutrition knowledge, learned how to read a nutrition label, and picked up healthy snack alternatives. They also provided students with snack recipes that could be made in the residence halls and on a low budget.

### Cruising for Losing

This activity was held over 2 days and consisted of the group members holding different group fitness workouts and giving a presentation to participants on the importance of physical activity.

## Reflections

To show the outcomes of active learning from a student and instructor perspective, comments students gave during their final presentation were used to provide overall student reflections. Instructor reflections are based on each author’s view of how using active learning impacted student outcomes.

### Student Reflections

During group presentations of their health activity, students provided reflections on what they learned about health program planning. Overwhelmingly, most students commented on how hard it was planning their activity because it required more work than they imagined. Many stated how they planned for things to go one way, but when they implemented their activity, things went another way. When things went in a different direction, they weren’t as prepared to execute their activity effectively. They also stated that working in a group was sometimes difficult because they had to learn how to work together and come to an agreement on what to do.

In early sections of this course, many students stated that they would have liked to collect their own data with their target population for their needs assessment instead of using secondary data results from organizations like the Centers for Disease Control and Prevention. Due to the need for IRB approval for data collection and the lag time that may take place with getting IRB approval at the university, students have only used secondary sources to develop their needs assessment. Having students collect their own data would give them skills and experience in surveying a population.

### Instructors’ Reflections

After teaching this course, there are some elements about the course that were beneficial as an instructor. Allowing students the opportunity to practice speaking in front of a crowd with their health presentation and skill demonstration, gave them an opportunity to get feedback on what did and did not work with their presentation. This level of feedback had a noticeable improvement on each student’s presentation skills. Students were nervous doing their health presentation but after getting feedback, they were more comfortable doing the skill demonstration exercise. Another element that was beneficial to the course was scheduled class time to work on the class project.

Because planning a health activity involves a lot of steps, allowing students the opportunity to work in their groups on each assignment during class gave them an opportunity to ask the instructors for feedback on things they were considering or struggling with. Additionally, having the whole class work through developing goals and objectives for a general health issue like heart disease gave them additional exposure to elements they needed to plan their health activity. Both of these elements allowed students to have a better understanding of the assignments and what they were being asked to do.

## Discussion

In this article, we discussed our approach to teaching a health promotion course to undergraduate students at a HBCU using assignments that required students to be engaged in active learning. We believe engaging the students this way with these activities gave them the opportunity to learn the material in a more meaningful way because they had to use their classroom knowledge to develop a health activity that their peers would be engaged in. After teaching this course, we believe some elements of how this course was developed could be replicated for other health education or health program courses. To provide teachers a guide on how to include active learning assignments within their course, we have provided some lessons learned.

### Lessons Learned

- *Planning for group dynamics*: as instructors, we assign group work so students know how to work in groups, but we don’t take the time to explain to them how to work in groups effectively. What might be helpful for a course like this is to talk about how to work in a group in a professional manner at the beginning of the course so that students have a better understanding of what they need to do. This may help with them understanding that they can’t work individually or switch to another group because they’re not getting along with their group members. It may also be beneficial to provide students with structure on how to select group members based on characteristics such as personality and academic strengths, which may also help reduce conflicting group dynamics.
- *Making assignments feasible*: because we wanted students to develop and implement a health program, the assignments they were given had to be things that they could do in a 15- or 16-week semester course. For this course, students developed one goal, one objective, and one program activity. By having students only develop one goal, objective, and activity, they had a project that they were able to develop and implement within one semester. For other health courses, instructors should pick one or two skills or outcomes that you want students to have at the end of the course and plan assignments around that. This may give you more time to spend on assignments that will develop more skills in students instead of giving them a lot of assignments that don’t allow them to develop and retain the information or skill.
- *Covering course material early*: since students need to implement their program by the fourth month of the semester, course material had to be covered pretty early so they could turn in assignments and receive feedback from the instructor before implementing their activity. Covering material early also allowed for some flexibility with assignment dates if things needed to be moved around. For other health courses, if you decide that you want students to have one major project at the end, covering course material early will also allow you flexibility toward the end of the course with assignments and will allow you to help students with assignments that they find difficult.
- *Having assignments in pieces*: because students are being asked to plan and implement a health program, turning in assignments in pieces made it easy to monitor the program development process, so errors could be corrected early. This also helped with determining if some assignment dates needed to be moved, changes needed to be made to assignment guidelines, or more class time needed to be provided for groups to work on assignments. The lesson works for any course, having students turn in assignments in pieces makes easier grading for the instructor, and gives students a chance to fix errors that they have before they turn in the final document. Having assignments set up this way also allows the instructor to know if students understand the information and what information you may need to go back over in class.
- *Giving opportunity to practice health education skills*: since many students are nervous about implementing a health activity where they are the ones giving the information, giving them an opportunity to practice delivering health information is important. Having these assignments in the course allow students to become comfortable speaking in front of a group and it gives them practice on how to engage their audience. For other health course skills, instructors should plan assignments where students have to practice the skill in front of the class or another audience. For example, with a nutrition course, you may find it beneficial to have students do a mock health or food assessment in the class if you are going to have them do one as a final assignment. For a health policy course, if you are going to have students advocate for a local ordinance or national bill at city hall, the state capital, or Washington, DC; it may be beneficial to make them practice their advocacy skills in front of the class as an assignment so that you can give them feedback and so they are prepared before the event occurs.
- *Remaining current*: because elements of health programming are always changing, assignments in the class need to reflect these changes. For instance, the social media blurbs for the press kit were added because social media has become a large platform and most college students give and receive information using these platforms. Requiring students to develop social media blurbs helped them think about how to use social media effectively when planning a program. With other health course, remaining current in your course material means that students will stay up to date on skills they need once they graduate. For instance, within a nutrition course, you need to make sure that your assignments are continually reflective of updated dietary guidelines. For courses related to environmental issues or statistics, you want to make sure that your assignments reflect current issues, current data trends, or new data analysis.
- *Working with health organizations*: because this type of course seeks to develop health education skills, it would be a good fit to try and make this a service-learning course by having the class work with student health services or local health organizations to develop and implement health activities or programs that would be beneficial to these groups. Many small campuses may not have health programming on campus, so a health program course could fill student health programming gaps. Connecting the course with a group on campus or in the community makes things easier because the instructor and students know what they need to do at the beginning of the course. This also gives students real-world experience in working with stakeholders to meet their organization’s needs along with needs of the target population. Using service-learning with community organizations or campus wellness programing for this course provides students with an opportunity to develop skills necessary for problem solving while also helping them address community issues (10). For other health course, you don’t have to include outside organizations or groups in your class, but you can develop assignments that will mimic real-world experiences for your students so that they are engaged in the course while developing necessary skills for their field.
- *Involving students*: typically, students do not provide input on the class assignments. However, asking students at the beginning of the course if they want to work on a class health program with groups doing different parts of the program or work on health activities separately might make students more engaged in the class project and allow them to take more ownership on the work they are doing. If the students decide that they want to work on one health program, true implementation of a full health program may be feasible. For other health courses, if you are planning to do a culminating class assignment, it may be beneficial to propose two assignment working options for students at the beginning so that adjustments can be made early and so students are more engaged in the work that will be required for the course.

## Conclusion

With the number of bachelor of public health programs continually increasing, it is important to understand how these programs are preparing their students to be active and engaged public health professionals. Therefore, there needs to be more research on teaching pedagogies that prepare undergraduate public health students to enter the public health workforce upon graduation. While the outcomes of using active learning are mixed, this pedagogical style speaks to the notion of making sure that students are engaged in the material they are learning so that they are able to use it outside of the classroom (7–9). Although there may be more effective pedagogical frameworks, styles, or methods; more research needs to be done on which pedagogical frameworks are more effective at preparing students to address any future public health issue or crisis. With the growth of undergraduate public health programs, public health can’t continue to teach in the same manner to prepare students for the future. A recent article on Harvard’s School of Public Health changing their teaching to promote continuous learning in their students is an example that because public health is always evolving, we as teachers and instructors must always evolve in how we teach the next generation of public health professionals (11).

## Author Contributions

KM and TG conceptualized the manuscript together and each provided their reflection on the teaching of the course and lessons learned. KM wrote the introduction and both authors contributed to all other sections.

## Conflict of Interest Statement

The authors declare that the research was conducted in the absence of any commercial or financial relationships that could be construed as a potential conflict of interest.
